# Food Effects, Pharmacokinetic Drug–Drug Interactions, and Clinical Optimization of Oral Anticancer Agents

**DOI:** 10.3390/pharmaceutics18091082

**Published:** 2026-08-28

**Authors:** Abdullah A. Assiri

**Affiliations:** Department of Clinical Pharmacy, College of Pharmacy, King Khalid University, Abha 61421, Asir, Saudi Arabia; aalabdullah@kku.edu.sa

**Keywords:** oral anticancer agents, pharmacokinetics, food effect, drug–drug interactions, CYP3A4, P-glycoprotein, acid-suppressive therapy, oncology pharmacy, therapeutic drug monitoring

## Abstract

Oral targeted therapies now constitute a substantial and growing proportion of anticancer drug therapy, shifting administration from the controlled intravenous setting to patient-managed oral therapy in the outpatient setting, where systemic exposure depends on factors that parenteral therapy largely bypasses. Food effects, gastric pH, first-pass metabolism, transporter activity, organ function, concomitant medications and adherence contribute to variability in exposure, and many oral anticancer agents have narrow therapeutic indices in which modest exposure changes carry clinical consequence. This review synthesizes the pharmacokinetic determinants of oral anticancer drug exposure across twenty-seven exemplar agents spanning the main mechanistic classes and translates them into actionable pharmacy practice. Its contribution is a cross-class agent-level comparison in which within-class divergences are made explicit, the integration of determinants usually reviewed separately, and an explicit statement of the evidence level behind every entry. We examine food effects, the interaction between acid-suppressive therapy and pH-dependent agents, the dominant role of cytochrome P450 3A4 and of the efflux transporters P-glycoprotein and breast cancer resistance protein, and the exposure–response relationships that motivate therapeutic drug monitoring, for which the evidence remains uneven and does not yet support routine use. We propose a structured framework for operationalizing these principles in daily practice.

## 1. Introduction

The therapeutic landscape of oncology has changed markedly with the proliferation of orally administered targeted agents [1,2]. Tyrosine kinase inhibitors (TKIs), cyclin-dependent kinase 4/6 (CDK4/6) inhibitors, poly(ADP-ribose) polymerase (PARP) inhibitors, Bruton tyrosine kinase (BTK) inhibitors, and B-cell lymphoma 2 (BCL-2) inhibitors, among others, have moved a meaningful proportion of cancer treatment out of the infusion suite [2,3]. Oral administration confers obvious advantages in convenience, autonomy, and resource utilization, but it transfers responsibility for correct dosing and timing to the patient and introduces a set of pharmacokinetic vulnerabilities that intravenous therapy does not face [3,4].

Intravenous administration delivers a known quantity of drug directly to the systemic circulation, bypassing the absorptive and presystemic processes that govern oral bioavailability. By contrast, an orally administered agent must dissolve in gastrointestinal fluid, remain in solution across a fluctuating luminal pH, permeate the intestinal epithelium against the activity of efflux transporters, and survive first-pass extraction in the gut wall and liver before reaching the systemic circulation. With the shift from intravenous to oral administration, absorption becomes a newly introduced and often decisive factor in drug disposition [1,2]. Many oral anticancer agents are poorly water-soluble weak bases whose dissolution depends on gastric acidity; many are substrates of cytochrome P450 (CYP) 3A4 and of the efflux transporters P-glycoprotein (P-gp) and breast cancer resistance protein (BCRP); and many display steep relationships between systemic exposure and either antitumor effect or dose-limiting toxicity.

These properties make oral anticancer therapy uniquely susceptible to clinically meaningful perturbation. A meal can alter bioavailability several-fold for some agents. Concurrent acid-suppressive therapy—frequently prescribed and often initiated without reference to the oncology regimen—can substantially reduce the absorption of pH-dependent drugs [5]. Co-administration of a strong CYP3A4 inhibitor or inducer can shift exposure to a degree that warrants dose modification or avoidance. Hepatic impairment, advanced age, polypharmacy, and imperfect adherence further widen the range of achievable exposures. Because the consequences of these perturbations fall on a narrow therapeutic margin, the difference between optimal and suboptimal management is often pharmacokinetic rather than pharmacodynamic.

Several reviews have addressed individual components of this problem, including food effects and acid-reducing-agent interactions in oral targeted therapy [1], the bioavailability and drug–drug interaction potential of protein kinase inhibitors [2], and therapeutic drug monitoring of oral anticancer agents [6]. What is less well served is an account that holds these determinants together at the level of the individual agent, so that a clinician facing a specific prescription can see in one place how food, gastric pH, cytochrome P450 and transporter interactions, organ function and adherence combine for that drug, and how strong the evidence for each of those statements is. The novelty claimed for the present review is therefore threefold and deliberately practical. The review provides a cross-class, agent-level comparison of twenty-seven exemplar oral anticancer agents in which within-class divergences are made explicit rather than absorbed into class statements. It integrates determinants that are usually reviewed separately into a single exposure narrative and records, for every agent-level entry, whether the statement rests on a dedicated clinical pharmacokinetic study, on labeling, or on mechanistic inference. It then converts these principles into a structured, cyclical optimization framework that can be applied at the point of care. No new experimental data are presented, and the contribution should be judged as synthesis and translation rather than as discovery.

The purpose of this review is to consolidate the pharmacokinetic principles most relevant to oral anticancer agents and to render them clinically actionable for the oncology pharmacist. We focus deliberately on absorption and disposition phenomena—food effects, gastric pH and acid suppression, CYP- and transporter-mediated interactions, exposure–toxicity relationships, and special-population considerations—and we conclude with a practical framework for pharmacokinetic optimization in routine practice. The review is scoped to oral targeted and hormonal agents in which pharmacokinetics drive clinical decisions about dose, scheduling, and concomitant therapy, rather than as a comprehensive survey of oncology pharmacology.

## 2. Methodological Approach and Literature Identification

This review is narrative rather than systematic in design, and the literature underpinning it was identified by searching PubMed/MEDLINE, Scopus and Web of Science for English-language publications, supplemented by Embase where accessible, from database inception to a search cut-off date of 1 June 2026. All regulatory sources were retrieved or re-verified on that date, which is recorded as the access date for every prescribing information document and summary of product characteristics in the reference list. No further update window was applied, so agents approved after that date are not represented, and the consequences of that boundary are discussed in Section 11. Search terms combined drug-class and individual-agent names with pharmacokinetic descriptors, including “oral anticancer agents,” “tyrosine kinase inhibitor,” “food effect,” “pharmacokinetics,” “bioavailability,” “AUC,” “Cmax,” “CYP3A4,” “P-glycoprotein,” “BCRP,” “drug interaction,” “acid-reducing agents,” “proton pump inhibitors,” “gastric pH,” “hepatic impairment,” and “therapeutic drug monitoring.”

Primary regulatory sources were consulted directly: United States Food and Drug Administration (FDA) prescribing information, and European Medicines Agency (EMA) summaries of product characteristics where they added clinically relevant nuance. Where a pharmacokinetic value or administration recommendation is reported, the originating label or primary study was retrieved and read rather than relied upon secondarily. Priority was given to primary clinical pharmacokinetic studies, dedicated food-effect and drug-interaction studies, official labeling, and authoritative clinical pharmacology reviews; systematic reviews and meta-analyses were incorporated where available. Where sources disagreed, a fixed hierarchy was applied. Quantitative pharmacokinetic values, that is, percentage or fold changes in the area under the concentration–time curve (AUC) and the maximum concentration (Cmax), were taken preferentially from the dedicated clinical study reporting them, whether published independently or reported quantitatively in the clinical pharmacology section of the approved label. Administration and management recommendations were taken from the current FDA prescribing information, because that is the document that governs practice in the setting for which the recommendation is made. The EMA summary of product characteristics was cited in addition wherever it reaches a materially different conclusion, rather than the divergence being silently reconciled; the differing US and European positions on the esomeprazole–lapatinib interaction (Section 6) are reported in this way. Where a label states only a predicted or modelled effect, that status is preserved in the text and in Table 1, Table 2 and Table 3 rather than being reported as a measured value. We did not perform a formal systematic review with a registered protocol, predefined inclusion and exclusion criteria, and reproducible screening; this work should not be interpreted as such.

This work profiles twenty-seven oral targeted and hormonal anticancer agents, chosen as a mechanistic illustration rather than as a population sample, since the more than one hundred currently approved oral anticancer agents include numerous within-class analogues whose pharmacokinetic profiles closely parallel those already represented here. Agents were included when they satisfied the first of the following criteria and at least one of the remaining four. (i) They represent a major mechanistic class in current oral oncology practice, namely tyrosine kinase, cyclin-dependent kinase 4/6, poly(ADP-ribose) polymerase, Bruton tyrosine kinase, B-cell lymphoma 2, BRAF/mitogen-activated protein kinase kinase (MEK), anaplastic lymphoma kinase (ALK), mammalian target of rapamycin (mTOR) or androgen-axis inhibition. (ii) A dedicated food-effect, acid-reducing-agent, drug-interaction or organ-impairment study is available, published independently or reported quantitatively in the approved labeling, so that the agent can be described with a value rather than an adjective. (iii) The labeled administration or interaction instruction is of sufficient magnitude to change clinical management, such as a mandated fasting condition, a contraindicated co-medication or a specified dose reduction. (iv) The agent diverges from the expectation set by its own class, which is the property that makes agent-level rather than class-level review necessary, the clearest instances being acalabrutinib against ibrutinib for acid suppression and apalutamide against enzalutamide for the magnitude of the CYP3A4-inducing effect, both of which are discussed in the relevant sections. (v) The agent is in sufficiently broad clinical use for the resulting set to be recognizable to a practitioner managing oral anticancer therapy. Agent-by-agent food-effect, drug-interaction, and organ-impairment data are provided in Table 1, Table 2 and Table 3, and Section 5, Section 6, Section 7 and Section 8 illustrate each pharmacokinetic phenomenon with the agents that exemplify it. The boundaries of this sample and the principal post-2023 exclusions are discussed in Section 11.

Because a narrative review draws on sources of unequal strength, every agent-level entry in Table 1, Table 2 and Table 3 carries an explicit evidence descriptor. The pharmacokinetic (PK) descriptor denotes a value derived from a dedicated clinical pharmacokinetic study in humans, whether a food-effect, acid-reducing-agent, drug-interaction or organ-impairment study, for which a quantitative or study-derived result is reported in the table. L denotes a labeled instruction, dose adjustment or labeled conclusion of no clinically significant effect that is reported without an accompanying quantitative result. M denotes a mechanistic property or a model-based prediction that has not been quantified in an administered clinical study, and M/PK marks an entry that combines both. These descriptors grade the basis of the entry rather than its clinical importance, and an M entry may carry greater practical weight than a PK entry. Two consequences are applied throughout. An L entry does not imply that no dedicated study exists, and the absence of an interaction from prescribing information is recorded as the absence of a labeled finding rather than as evidence that no interaction occurs, particularly where the agent is a known substrate, inhibitor or inducer of cytochrome P450 enzymes or clinically relevant transporters [7]. Conversely, mechanistic plausibility alone is never presented as a quantified clinical interaction.

The limitations that follow from this design, including the element of judgement involved in agent selection and the absence of a formal risk-of-bias appraisal, are set out together with the other limitations of the review in Section 11.

## 3. Evolution of Pharmacokinetic Management of Oral Anticancer Agents

The recommendations summarized in this review are not a static body of knowledge but the product of roughly two decades of accumulating clinical experience, regulatory expectation and formulation science, and several of the instructions that now appear routine were absent when the first oral targeted agents entered practice. When imatinib was introduced, the pharmacokinetic instruction attached to an oral anticancer agent was minimal: bioavailability is essentially complete and food is relevant chiefly to gastrointestinal tolerability rather than to exposure, so administration guidance amounted to taking the dose with food and water [8]. The agents that followed were, on the whole, less forgiving. As development moved toward lipophilic, poorly soluble, weakly basic kinase inhibitors, the properties that made these molecules potent also made their absorption conditional, and the instruction attached to a prescription lengthened accordingly—from a tolerability note to mandated fasting windows, contraindicated co-medications and defined dose reductions [2].

Two vulnerabilities were recognized in sequence, and the order in which they were recognized still shapes how well each is handled in practice today. Food effects were characterized first and were initially treated as a nuisance variable to be standardized within registration studies rather than as a clinical lever, and the observation that a meal could alter exposure several-fold, and in opposite directions for different agents, moved them into the category of information that must be transmitted to the patient [1,9]. The interaction between acid-reducing agents and pH-dependent weak bases was recognized later, and it remained underappreciated in practice for some time after it had been established in principle, as Section 6 describes. The regulatory expectation followed the science, and the design and interpretation of gastric pH-dependent interaction studies is now the subject of its own guidance to industry [10].

The systematic assessment of drug–drug interactions during development followed a similar trajectory. Earlier labels frequently described interaction potential qualitatively, in terms of enzyme and transporter designations, whereas current labels for the agents reviewed here typically report the measured effect of a strong index inhibitor and of a strong index inducer, expressed as a fold or percentage change in exposure, together with the dose adjustment that follows from it. That change reflects a deliberate, risk-based framework in which in vitro characterization triggers defined clinical studies [7], and it is the reason a review of this kind can now report values where a decade earlier it could report only mechanisms. The same maturation is visible in the emergence of the perpetrator question—whether the anticancer agent alters the disposition of the patient’s other medicines—which is now studied routinely and which, for enzalutamide and apalutamide, proves to be the dominant clinical issue [11,12].

Formulation science has repeatedly rewritten administration instructions without any change to the active molecule. Salt selection, amorphous solid dispersions, particle-size reduction and wetting agents have all been deployed to reduce dissolution-limited absorption [13,14], with the consequence that the administration instruction increasingly belongs to the product rather than to the drug, a distinction developed with worked examples in Section 5. Most recently, attention has begun to move from standardizing exposure to measuring it. Proposed concentration targets and structured protocols for monitoring in oral oncology [6], together with agent-specific evaluations for venetoclax and pazopanib [15,16], mark the beginning of exposure-guided rather than instruction-guided management, although the evidence supporting this last step remains uneven and, for most agents, preliminary, as Section 8 sets out. The sections that follow describe the state that this history has produced.

## 4. Pharmacokinetic Determinants of Oral Anticancer Drug Exposure

**Figure 1:**
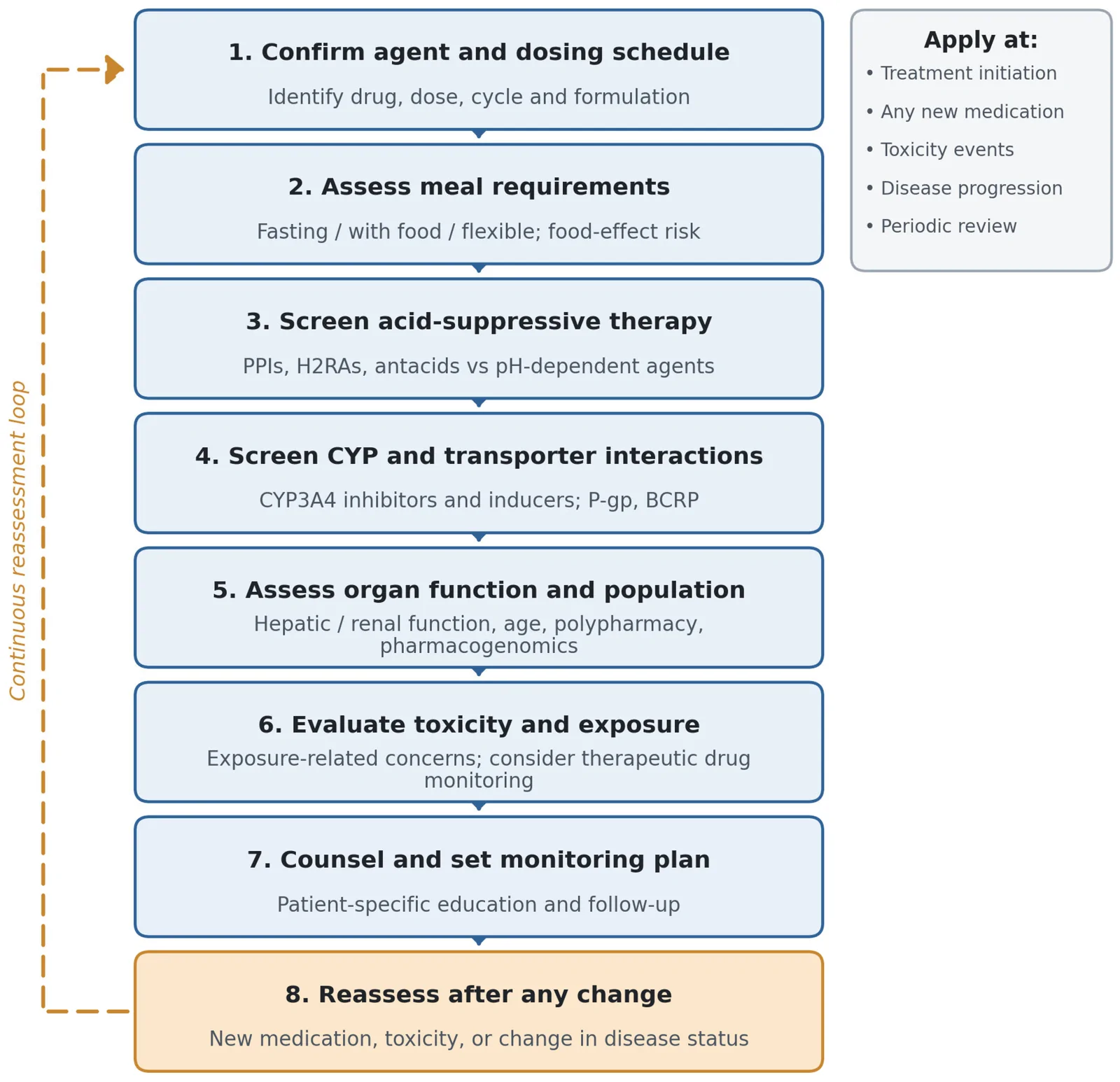
Clinical pharmacokinetic optimization framework for oral anticancer agents. The eight-step cyclical workflow is applied at initiation and revisited at each clinically relevant change. The dashed arrow indicates continuous reassessment. BCRP, breast cancer resistance protein; CYP, cytochrome P450; H2RA, histamine H2-receptor antagonist; P-gp, P-glycoprotein; PPI, proton pump inhibitor.

**Figure 2:**
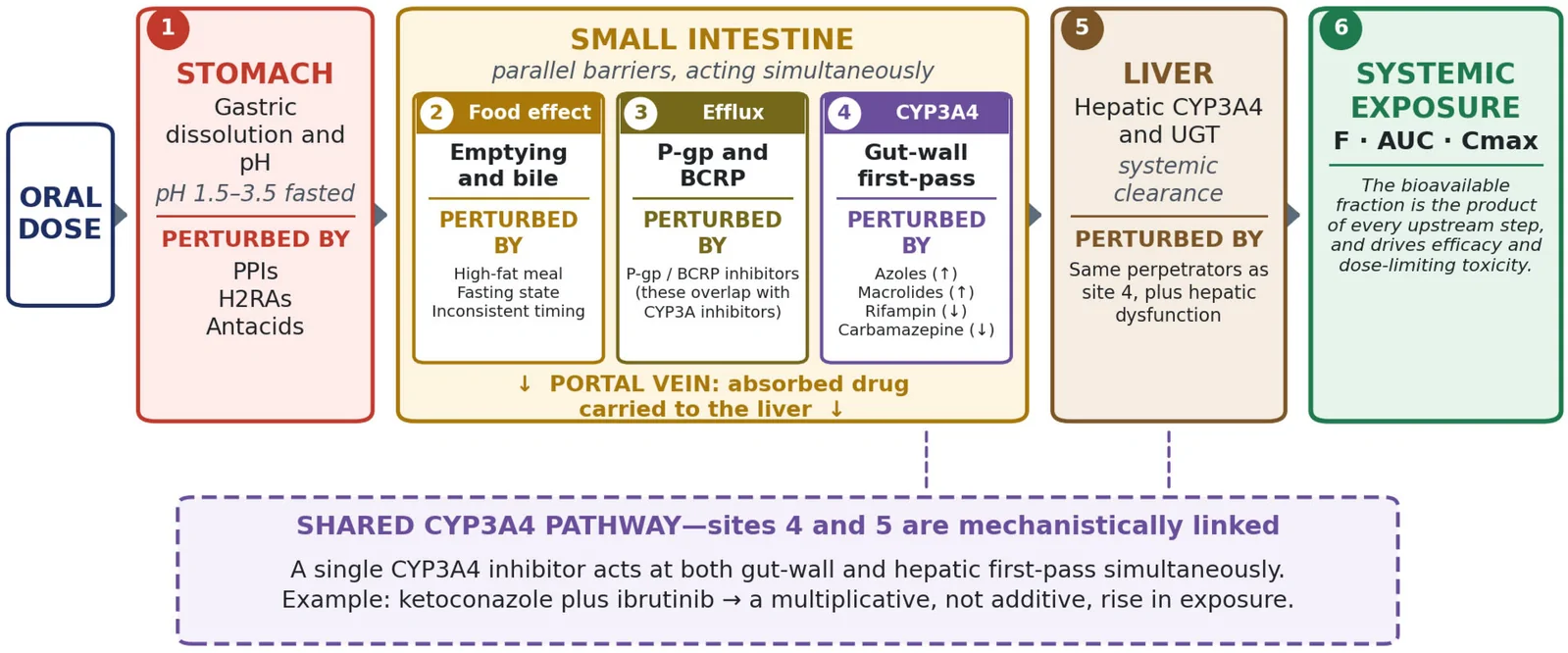
The absorption journey of an oral anticancer agent and where each barrier acts. Six numbered sites along the gastrointestinal–hepatic–systemic axis correspond to the principal points at which exposure can be perturbed: (1) gastric dissolution and pH; (2) food effect on emptying, bile secretion, and solubilization; (3) intestinal efflux by P-glycoprotein and BCRP; (4) enterocyte CYP3A4 first-pass; (5) hepatic CYP3A4 and UGT metabolism; and (6) the resulting systemic exposure (AUC, Cmax). Common interventions and their loci are annotated. Solid arrowheads between panels denote the sequential progression of the dose along that axis, and the dashed connectors link sites 4 and 5 to the shared CYP3A4 pathway; within site 4, ↑ and ↓ indicate perpetrators that increase and decrease exposure, respectively; the downward arrows flanking the portal-vein annotation indicate the direction of transfer of absorbed drug to the liver; and → denotes “leads to”. AUC, area under the concentration–time curve; BCRP, breast cancer resistance protein; Cmax, maximum concentration; CYP, cytochrome P450; F, oral bioavailability; H2RA, histamine H2-receptor antagonist; P-gp, P-glycoprotein; PPI, proton pump inhibitor; UGT, uridine diphosphate glucuronosyltransferase.

The systemic exposure achieved by an oral anticancer agent is the product of a sequence of processes, each governed by drug-specific and patient-specific factors [2]. The clinical workflow that operationalizes these determinants in practice is presented as Figure 1 (Section 10). Figure 2 schematizes these processes along the absorption journey and indicates where common interventions perturb each step. Understanding where a given agent is vulnerable within this sequence is the basis for anticipating and managing variability.

### 4.1. Solubility and Dissolution

Oral bioavailability begins with dissolution of the solid dosage form in gastrointestinal fluid. Many small-molecule oncology agents are lipophilic, poorly soluble compounds, and a substantial fraction behave as Biopharmaceutics Classification System (BCS) class II or class IV drugs in which dissolution rather than permeability limits absorption; notably, the BCS does not reliably predict the direction or magnitude of food effects for class II compounds [1]. For weakly basic compounds, solubility is pH-dependent and highest in the acidic environment of the fasted stomach, so loss of gastric acidity can reduce the fraction dissolved and available for absorption [17,18]. Formulation strategies—salt selection, amorphous solid dispersions, particle-size reduction, and wetting agents—are deployed to mitigate dissolution-limited absorption [13,14].

### 4.2. Gastric pH and Intestinal Transit

Luminal pH varies along the gastrointestinal tract and with prandial state and concomitant medication. For pH-sensitive agents, this variability translates directly into variable dissolution and absorption [18]. Gastric emptying and intestinal transit time determine the window during which a drug is presented to its absorptive sites; delayed emptying, as occurs after a meal, can alter both the rate and, for some agents, the extent of absorption [9].

### 4.3. Permeability and Intestinal Transporters

Once dissolved, a drug must cross the intestinal epithelium. Passive permeability is modulated by apically expressed efflux transporters—principally P-glycoprotein (P-gp, ABCB1) and breast cancer resistance protein (BCRP, ABCG2)—which return substrate to the lumen and limit net absorption [2]. Uptake transporters of the organic anion transporting polypeptide (OATP) family contribute to the hepatic uptake of certain substrates. Because many oral anticancer agents are substrates of these transporters, transporter-mediated interactions are a recurring mechanism of altered exposure [19].

### 4.4. First-Pass and Systemic Metabolism

Drug surviving the intestinal lumen is subject to metabolism in the enterocyte and, after portal absorption, in the liver. Cytochrome P450 3A4 (CYP3A4) is the dominant enzyme in the metabolism of oral anticancer agents, and CYP3A-mediated presystemic extraction is a principal determinant of bioavailability for many of them [2]. Additional pathways—including other CYP isoforms and, for some agents, uridine diphosphate glucuronosyltransferase (UGT)–mediated glucuronidation—contribute to specific drugs. The prominence of CYP3A4 explains why this enzyme is the single most important locus of pharmacokinetic drug–drug interactions in this therapeutic area.

### 4.5. Protein Binding, Hepatic Function, and Renal Contribution

Most oral anticancer agents are highly protein-bound, and only the unbound fraction is pharmacologically active and available for distribution, metabolism, and elimination. Because hepatic metabolism dominates the clearance of most of these agents, hepatic impairment is a frequent determinant of altered exposure and a common basis for dose adjustment. Renal elimination of unchanged drug is comparatively minor for many oral targeted agents, though renal contribution and the disposition of active metabolites are agent-specific.

**Figure 3:**
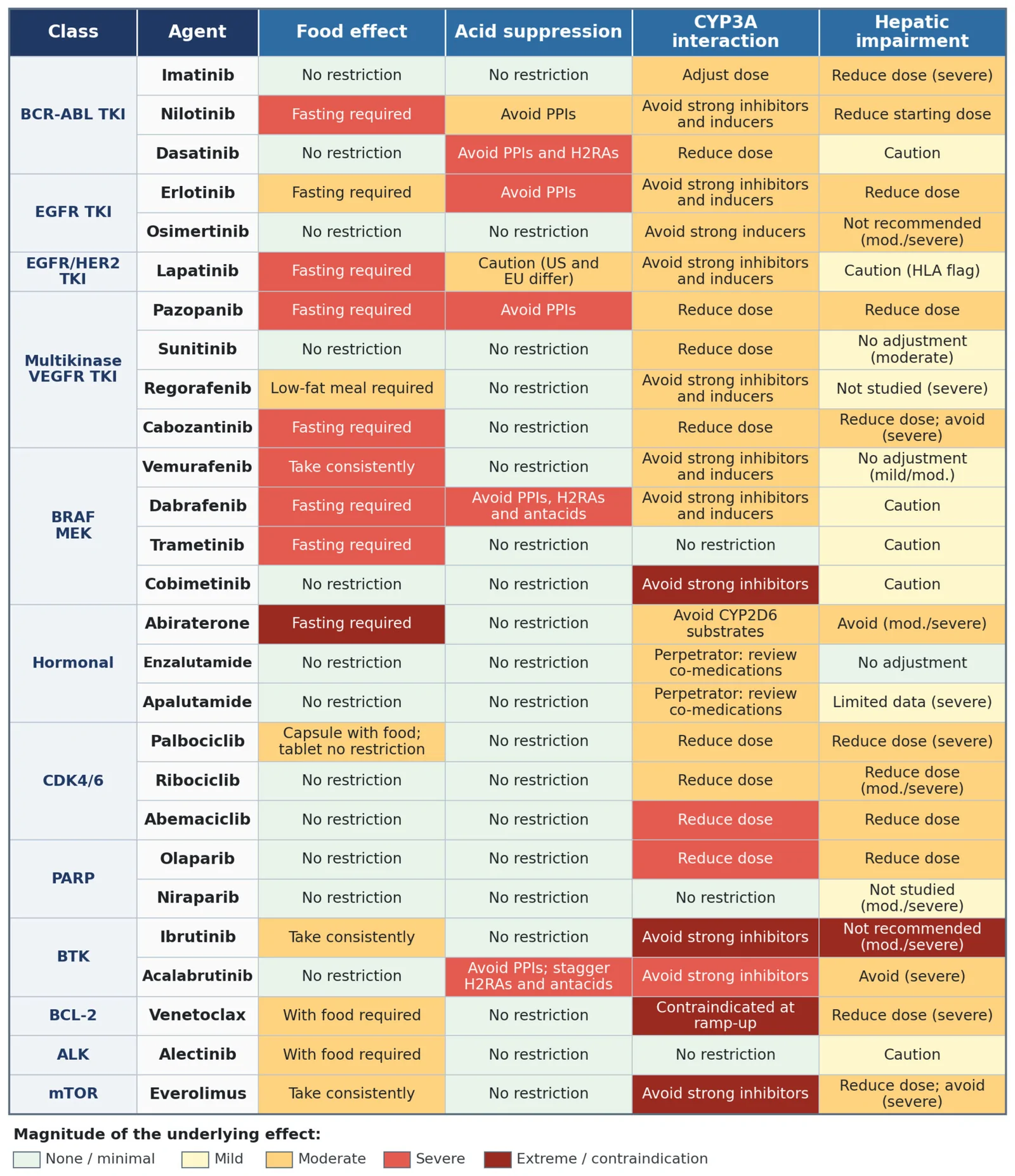
Pharmacokinetic vulnerability matrix for the twenty-seven exemplar oral anticancer agents, grouped by mechanistic class. Each cell states the clinical recommendation that applies to that agent on that axis, and color intensity reflects the magnitude of the label-documented vulnerability that lies behind the recommendation, across four axes: food effect, acid suppression, CYP3A interaction, and hepatic impairment. The five color categories were assigned as follows. None or minimal: no clinically significant effect reported on that axis and no action required. Mild: an effect or a gap in the data is noted but it does not by itself change the dose or the administration instruction, so that caution or monitoring is sufficient. Moderate: the effect changes management in a way that is routinely accommodated, such as an administration condition, a defined dose adjustment or staggering of doses. Severe: the prescription must be actively planned around the effect, whether through a mandated fasting condition, a recommendation to avoid the interacting agent where possible, or a large exposure change. Extreme or contraindication: co-administration is contraindicated or not recommended, or the exposure change is of an order that makes it unmanageable. Assignment was made separately for each axis and combines the magnitude of the reported pharmacokinetic change with its clinical management consequence, the category reflecting the greater clinical consequence being chosen where the two diverged. The scale is ordinal, is not a numerical threshold rule, and is intended for orientation only. “No restriction” indicates that no dose or administration change is required, not that an interaction has been excluded. The quantitative values, the interacting agent, and the level of evidence supporting each entry are given in Table 1, Table 2 and Table 3, which are authoritative. The matrix pulls together the agent-level evidence detailed in Table 1, Table 2 and Table 3 and Section 5, Section 6 and Section 7 and reveals patterns that prose alone obscures, with multikinase TKIs and BRAF agents clustering on the food-effect axis, BCR-ABL TKIs and acalabrutinib on acid suppression, and ibrutinib, cobimetinib, and everolimus as the CYP3A extremes. Enzalutamide and apalutamide are flagged separately because their clinically dominant role is as CYP3A4 perpetrators (inducers) rather than victims. ALK, anaplastic lymphoma kinase; BCL-2, B-cell lymphoma 2; BCR-ABL, breakpoint cluster region–Abelson; BTK, Bruton tyrosine kinase; CDK4/6, cyclin-dependent kinase 4/6; CYP, cytochrome P450; EGFR, epidermal growth factor receptor; H2RA, histamine H2-receptor antagonist; HER2, human epidermal growth factor receptor 2; HLA, human leukocyte antigen; MEK, mitogen-activated protein kinase kinase; mTOR, mammalian target of rapamycin; PARP, poly(ADP-ribose) polymerase; PPI, proton pump inhibitor; TKI, tyrosine kinase inhibitor; VEGFR, vascular endothelial growth factor receptor.

Figure 3 summarizes how these determinants translate into agent-specific vulnerabilities across the twenty-seven exemplar agents profiled in this review, with the color gradient indicating the magnitude of the label-documented effect at each of four key axes: food, acid suppression, CYP3A interaction, and hepatic impairment; the rules by which each color category was assigned, and the fact that the scale combines the magnitude of the pharmacokinetic change with its clinical management consequence, are stated in the legend to Figure 3.

## 5. Food Effects on Oral Anticancer Agents

Food can alter the bioavailability of an oral anticancer agent substantially, and the direction and magnitude of the effect are agent-specific [1,9]. A meal changes the gastrointestinal environment in several ways that bear on drug absorption: it delays gastric emptying, stimulates biliary and pancreatic secretion, raises gastric pH transiently, increases splanchnic blood flow, and—particularly for a high-fat meal—provides lipid that can solubilize poorly water-soluble compounds and recruit them into mixed micelles. The net effect on a given drug depends on which of these mechanisms predominate.

For lipophilic, poorly soluble agents, the solubilizing effect of dietary fat frequently increases the fraction absorbed, sometimes markedly. The most striking example among the agents reviewed here is abiraterone acetate, for which the FDA label reports that a high-fat meal increased Cmax and AUC up to approximately 17-fold and 10-fold, respectively, relative to the fasted state [20]; because such an effect would render fed dosing both excessive and unpredictable, the label mandates administration on an empty stomach. Lapatinib shows the same logic at smaller magnitude: a low-fat breakfast increased lapatinib AUC by 167% and a high-fat breakfast by 325% versus fasting [21], and vemurafenib, although administered without regard to food in clinical trials, has an even larger labeled food effect (high-fat meal: AUC ↑ ~5-fold, Cmax ↑ ~2.5-fold) [22]. Pazopanib doubles its exposure when taken with a meal and is dosed fasting [1,23]; erlotinib’s absolute bioavailability rises from roughly 60% fasted toward 100% with food, and it is taken fasting to standardize exposure [24]. Cabozantinib and dabrafenib are likewise dosed on an empty stomach (at least one hour before and at least two hours after a meal) to avoid food-driven over-exposure [25,26], as is trametinib, for which a high-fat meal reduced Cmax by 70% [27].

Not every food effect argues for fasting administration; several agents are taken with food precisely because food improves or stabilizes absorption, among them venetoclax is administered with a meal and water, with low- and high-fat meals increasing exposure relative to the fasted state [28,29], and alectinib is taken with food, showing an approximately 3.1-fold increase in combined alectinib-plus-active-metabolite exposure with a high-fat meal and improved gastrointestinal tolerability [30,31]. Regorafenib is unusual among these agents in requiring a specifically low-fat meal (less than 600 calories and less than 30% fat), because both high-fat meals and the fasted state yield lower exposure of the active metabolites than a low-fat meal [32]. Still other agents are essentially unaffected: abemaciclib, sunitinib, osimertinib, ribociclib, niraparib, enzalutamide, and apalutamide may all be taken without regard to meals [11,12,33,34,35,36,37,38]; dasatinib likewise has no clinically significant food effect; and olaparib—although food slows its absorption without changing total AUC—is given without regard to food [39,40]. Imatinib is conventionally taken with food and water to reduce gastrointestinal upset rather than to alter exposure (its bioavailability is 98% regardless of food) [8]. Everolimus shows a noteworthy nuance: food modestly decreases its exposure (high-fat meal ↓ AUC 22%, light-fat ↓ AUC 32%), but the practical recommendation is consistency—either always with food or always without [41]. Formulation can determine the instruction independent of the molecule: the palbociclib capsule must be taken with food, whereas the reformulated palbociclib tablet may be taken with or without food [42,43], and a reformulated nilotinib product (approved in 2024) carries no mealtime restriction in contrast to the fasting requirement of the original capsule [44,45]. Where a food effect is formulation-dependent, Table 1 identifies the product to which the entry applies, and the counseling point should be checked against the product actually dispensed.

Three administration patterns therefore emerge, and the distinction is clinically important because patient counseling differs categorically among them. Some agents must be taken fasting because food drives over-exposure or unpredictable exposure, others must be taken with food because food improves or stabilizes absorption or tolerability, and for the remainder the prandial state is flexible and the counseling message is consistency rather than a particular condition. Beyond the binary of fasting versus fed, the timing of the dose relative to a meal is itself a determinant of exposure, so counseling should specify the interval, not merely the prandial state [46]. The clinical hazard is not only the food effect itself but the gap between labeled instruction and real-world behavior; a retrospective cohort found that overnight fasting before lapatinib reduced toxicity relative to nighttime dosing [47]. Where a large positive food effect exists, deliberate administration with food has been proposed to permit dose (and cost) reduction, the so-called “value meal” concept [48,49], although the accompanying increase in variability tempers enthusiasm for the approach outside of trials. Table 1 summarizes the food-effect profile and administration recommendation for each agent reviewed.

One caveat applies to every cross-agent comparison made in this section and in Table 1. The food-effect studies from which these values derive differ in design—in study population (healthy volunteers or patients), dose, single-dose versus multiple-dose or steady-state conditions, the caloric and fat content of the test meal, the analyte measured (parent drug alone or parent plus active metabolite), and the pharmacokinetic endpoint reported. Descriptions such as “largest” or “most striking” in this review therefore rank magnitudes as they were reported in the respective studies and labels; they are not the output of a common experiment and should not be read as strictly comparable measurements. Such statements are offered to direct clinical attention rather than to establish a quantitative ordering among agents.

**Table 1:** Food-effect recommendations for the twenty-seven oral anticancer agents.

Drug	Class/Target	Effect of Food on Exposure	Recommended Administration	Clinical Recommendation	Evidence	Refs.
Imatinib	BCR-ABL TKI	No clinically significant effect	With food and water	Food taken to reduce GI upset, not to alter pharmacokinetics; oral bioavailability 98%	L	[8]
Nilotinib	BCR-ABL TKI	Food raises exposure and QT risk	Empty stomach: no food 2 h before, 1 h after	Fasting is in boxed warning; a reformulated product approved in 2024 has no meal restriction—check the product dispensed	L	[44,45,50]
Dasatinib	BCR-ABL TKI	No clinically significant effect	With or without food	Food timing not critical; acid suppression is the key issue	L	[39,51]
Erlotinib	EGFR TKI	F ~60% fasted to ~100% with food	Empty stomach: ≥1 h before/2 h after	Fasting standardizes exposure; smoking lowers it	PK	[24]
Osimertinib	EGFR TKI	Minimal (Cmax +14%, AUC +19%)	With or without food	Flexible; PPIs also have no effect on exposure	PK	[36]
Lapatinib	EGFR/HER2 TKI	Low-fat +167%; high-fat +325% AUC	Empty stomach: ≥1 h before/≥1 h after	Large, variable food effect; overnight fasting reduced toxicity in one cohort	PK	[21,47,52]
Pazopanib	VEGFR/multikinase TKI	Meal ~doubles AUC and Cmax	Empty stomach: ≥1 h before/2 h after	Fasting avoids food-driven over-exposure; do not crush	PK	[1,23]
Sunitinib	Multikinase TKI	No effect	With or without food	Flexible timing	L	[35]
Regorafenib	Multikinase TKI	Low-fat > high-fat > fasted (active metabolites)	With a low-fat meal (<600 cal, <30% fat)	Low-fat-meal requirement, uncommon among oral oncology agents	PK	[32]
Cabozantinib	Multikinase TKI	Food raises exposure	Empty stomach: ≥1 h before/2 h after	Strict fasting; tablets and capsules not interchangeable	L	[25]
Vemurafenib	BRAF inhibitor	High-fat meal: AUC ↑ 5×, Cmax ↑ 2.5×	With or without food, consistently	Large positive food effect; consistency > fasting/fed choice	PK	[22]
Dabrafenib	BRAF inhibitor	Food reduces absorption	Empty stomach: ≥1 h before/2 h after	Also avoid PPIs/H2RAs/antacids (pH-sensitive)	L	[26]
Trametinib	MEK inhibitor	High-fat: Cmax ↓ 70%, AUC ↓ 24%	Empty stomach: ≥1 h before/2 h after	Fasting required; large reduction in Cmax with a high-fat meal	PK	[27]
Cobimetinib	MEK inhibitor	No clinically meaningful effect	With or without food	Flexible; CYP3A is the key axis	L	[53]
Abiraterone	CYP17 inhibitor (hormonal)	High-fat: Cmax/AUC up to ~17×/10×	Empty stomach: no food 2 h before, 1 h after	Very large positive food effect; magnitude precludes fed dosing	PK	[20]
Enzalutamide	AR antagonist	No effect	With or without food	Concern is its perpetrator role on CYP3A substrates	L	[11]
Apalutamide	AR antagonist	No effect	With or without food	Like enzalutamide, a strong CYP3A inducer of co-meds	L	[12]
Palbociclib	CDK4/6 inhibitor	Tablet: no clinically significant effect. Capsule: exposure food-dependent	Tablet: any timing. Capsule: with food	Counsel by formulation; the two products differ in administration instruction	PK	[42,43]
Ribociclib	CDK4/6 inhibitor	No effect	With or without food	Flexible; QT monitoring required	L	[37]
Abemaciclib	CDK4/6 inhibitor	No clinically significant effect	With or without food	Flexible timing; continuous dosing	L	[33,34]
Olaparib	PARP inhibitor	Food slows abs. (Cmax ↓ 21%); AUC unchanged	With or without food	Tablets and capsules not interchangeable	PK	[40]
Niraparib	PARP inhibitor	No clinically significant effect	With or without food	Bedtime dosing may improve nausea tolerability	L	[38]
Ibrutinib	BTK inhibitor	Food: Cmax ~2–4×; AUC ~2×	With water, same time daily	Avoid grapefruit/Seville orange	PK	[54]
Acalabrutinib	BTK inhibitor	No clinically meaningful effect	With or without food	Food flexible; acid suppression is the major issue	L	[55]
Venetoclax	BCL-2 inhibitor	Low- and high-fat meals increase exposure versus the fasted state	With a meal and water	Always with food; pair with TLS ramp-up	PK	[28,29]
Alectinib	ALK inhibitor	High-fat: alectinib + M4 AUC 3.1×	With food	For bioavailability and GI tolerability	PK	[30,31]
Everolimus	mTOR inhibitor	High-fat ↓ AUC 22%/Cmax 54%	Consistently with or without food	Consistency matters more than the fasting/fed choice; TDM target 5–10 ng/mL (SEGA)	PK	[41]

AUC, area under the concentration–time curve; Cmax, maximum concentration; F, oral bioavailability; TKI, tyrosine kinase inhibitor; TLS, tumor lysis syndrome; PPI, proton pump inhibitor; H2RA, histamine H2-receptor antagonist; AR, androgen receptor; TDM, therapeutic drug monitoring; SEGA, subependymal giant cell astrocytoma; ALK, anaplastic lymphoma kinase; BCL-2, B-cell lymphoma 2; BCR-ABL, breakpoint cluster region–Abelson; BTK, Bruton tyrosine kinase; CDK4/6, cyclin-dependent kinase 4/6; CYP, cytochrome P450; CYP17, steroid 17α-monooxygenase; EGFR, epidermal growth factor receptor; GI, gastrointestinal; HER2, human epidermal growth factor receptor 2; MEK, mitogen-activated protein kinase kinase; mTOR, mammalian target of rapamycin; PARP, poly(ADP-ribose) polymerase; VEGFR, vascular endothelial growth factor receptor. ↑, increase; ↓, decrease. Evidence descriptors: PK, value derived from a dedicated clinical pharmacokinetic study in humans, reported here as a quantitative or study-derived result; L, a labeled instruction, dose adjustment or labeled conclusion reported without an accompanying quantitative result; M, a mechanistic property or model-based prediction that has not been quantified in an administered clinical study (M/PK indicates an entry that combines both). An L entry does not imply that no dedicated study exists, and the absence of an effect from labeling is recorded as the absence of a labeled finding rather than as evidence that no effect occurs. The descriptors grade the basis of the entry, not its clinical importance; see Section 2. Where a food effect is formulation-dependent, the formulation to which the entry applies is identified in the table.

## 6. Gastric pH, Acid-Suppressive Therapy, and Absorption

A majority of orally administered, molecularly targeted anticancer agents are weak bases that exhibit pH-dependent solubility, such that suppression of gastric acidity can impair their absorption—a vulnerability that has been described as a potential “Achilles heel” of targeted therapy [17,18]. The clinical relevance of this mechanism is amplified by epidemiology: acid-reducing agents—proton pump inhibitors (PPIs), histamine H2-receptor antagonists (H2RAs), and antacids—are among the most commonly used medications in the oncology population, and they are frequently taken without the prescriber’s or patient’s awareness of an interaction with cancer therapy [5,56].

The three classes differ in the magnitude, duration, and timing of their effect on gastric pH, and these differences shape management [10]. PPIs produce profound and sustained acid suppression that persists across the dosing interval; because their effect is prolonged, temporal separation from the anticancer agent is generally ineffective. The dasatinib label illustrates the hierarchy clearly: famotidine reduced dasatinib AUC by approximately 61% and omeprazole by approximately 43%, leading the label to advise against concomitant H2RAs and PPIs while permitting antacids if separated by at least two hours [39,51]. Erlotinib shows a comparably large PPI effect (omeprazole reduced AUC by 46% and Cmax by 61%), and its label advises avoiding PPIs where possible [24]. Nilotinib (esomeprazole reduced AUC by approximately 34%) and pazopanib (esomeprazole reduced exposure by approximately 40%) follow the same pattern [23,44,50].

Two contrasts within the available evidence are instructive, and both caution against generalizing from class membership. First, an agent may be pH-dependent in vitro yet show little clinically meaningful interaction in vivo: the palbociclib label reports that rabeprazole, under fed conditions, reduced Cmax by 41% but AUC by only 13% [43]. Second, regulatory agencies may reach different conclusions on the same agent: the US lapatinib label states that esomeprazole did not produce a clinically meaningful reduction in steady-state exposure, whereas the European summary of product characteristics reports an average 27% reduction (range 6–49%) that diminishes with increasing age [52,57]. Alectinib, by contrast, shows no clinically meaningful effect of esomeprazole on combined alectinib-plus-metabolite exposure, exemplifying an agent whose disposition is relatively insensitive to gastric pH [31,58]. A pharmacologically grounded mitigation has also been studied: co-administration of erlotinib with the acidic beverage cola increased erlotinib AUC by 39% in patients receiving esomeprazole [59].

A particularly illustrative contrast within a single class is acalabrutinib versus ibrutinib. Ibrutinib has no clinically significant acid-suppression interaction in its label, reflecting absorption that is not gated by gastric pH [54]. Acalabrutinib—a structurally distinct second-generation BTK inhibitor approved for similar indications—has solubility that decreases with rising pH, with the result that the PPI omeprazole reduces acalabrutinib AUC by 43%, calcium-carbonate antacid reduces AUC by 53%, and the label recommends avoiding concomitant PPIs entirely while staggering H2RAs and antacids by at least two hours [55]. Two drugs against the same target, in the same diseases, with categorically different acid-suppression instructions—an instructive lesson in why class membership does not predict pharmacokinetic behavior and why agent-level review is necessary at every initiation. Dabrafenib similarly recommends avoiding PPIs, H2RAs, and antacids because of pH-dependent solubility, despite belonging to a class (BRAF inhibitors) not traditionally associated with acid-suppression interactions [26]. By contrast, apalutamide is not ionizable across the physiologic pH range, so acid-reducing agents are not expected to affect its absorption [12]; the same is true of enzalutamide, so for this class the clinically decisive property is not pH sensitivity but the perpetrator role discussed in Section 7. This is itself a mechanistic rather than a studied conclusion, and it is recorded as such in Table 2. Management strategies follow a hierarchy of avoidance or substitution, temporal separation (effective for antacids and sometimes H2RAs, generally not for PPIs), and monitoring.

Three different kinds of statements appear in this section and in Table 2, and they should not be read as equivalent. A demonstrated clinical interaction rests on a dedicated study in which an acid-reducing agent was co-administered and the resulting change in exposure was measured, as for dasatinib, erlotinib, nilotinib, pazopanib and acalabrutinib. A labeled management recommendation may be more conservative than any single measured value, because it also reflects the therapeutic margin of the agent and the duration of acid suppression achieved by the class of acid-reducing agent involved. A mechanistic expectation derives instead from the ionization and solubility behavior of the molecule, as for apalutamide and niraparib. The absence of a labeled acid-suppression interaction is therefore recorded in Table 2 as the absence of a labeled finding, and not as evidence that no interaction exists, which matters particularly for weak bases in which pH-dependent solubility has not been formally studied. One further distinction carries more weight here than elsewhere in this review. Cytochrome P450 and transporter interactions are largely properties of the active molecule, whereas the magnitude of an acid-suppression interaction depends on dissolution behavior and therefore on the salt form, the solid-state form and the excipients of the particular product studied, so each value applies to the formulation examined in the cited source and should not be transferred without qualification to a different product of the same molecule [10]. Table 2 summarizes the acid-suppression profile for each agent.

**Table 2:** Acid-suppressive therapy interactions with the twenty-seven oral anticancer agents.

Drug	pH-Dependent	Interaction with PPIs/H2RAs/Antacids	Practical Implication	Clinical Recommendation	Evidence	Refs.
Imatinib	No (not primary)	No interaction reported in labeling; not formally evaluated	CYP3A is the relevant axis	None specific	L	[8]
Nilotinib	Yes	Esomeprazole (PPI) ↓ AUC ~34% (original capsule formulation)	PPI use materially lowers exposure	Short-acting antacids or H2RAs instead of PPIs	PK	[44]
Dasatinib	Yes	Famotidine ↓ AUC ~61%; omeprazole ↓ ~43%; antacid ↓ ~55%	Among the largest acid-suppression interactions reported for these agents	H2RAs/PPIs not recommended; antacids staggered ≥2 h	PK	[39,51]
Erlotinib	Yes	Omeprazole ↓ AUC 46%/Cmax 61%; ranitidine ↓ AUC 15–33%	Cola raised AUC 39% during esomeprazole	Avoid PPIs; if H2RA needed, take erlotinib 10 h after/≥2 h before	PK	[24,59]
Osimertinib	No	Omeprazole had no effect on exposure	Insensitive to gastric pH	None specific	PK	[36]
Lapatinib	Yes	US: no clinically meaningful ↓; EU: ~27% ↓ (range 6–49%)	US/EU labeling divergence	Caution with acid-reducing agents	PK	[52,57]
Pazopanib	Yes	Esomeprazole ↓ exposure ~40%	Common co-prescription that lowers exposure	Avoid concomitant PPIs where possible	PK	[23]
Sunitinib	No	No clinically significant interaction reported in labeling	CYP3A is the relevant axis	None specific	L	[35]
Regorafenib	No (not primary)	No interaction reported in labeling; not formally evaluated	Food-effect (low-fat) is the key axis	None specific	L	[32]
Cabozantinib	No (not primary)	No interaction reported in labeling; not formally evaluated	CYP3A and fasting are the key axes	None specific	L	[25]
Vemurafenib	No (not primary)	No interaction reported in labeling; not formally evaluated	Food effect dominates	None specific	L	[22]
Dabrafenib	Yes	Avoid PPIs, H2RAs, antacids	Within-class divergence from other BRAF inhibitors	Avoid concomitant acid-reducing agents	L	[26]
Trametinib	No	Not metabolized via gastric pH-dependent pathways	Hydrolytic esterase metabolism; pH-insensitive	None specific	M	[27]
Cobimetinib	No	Rabeprazole had no clinically significant effect	CYP3A is the dominant axis	None specific	PK	[53]
Abiraterone	No (not primary)	No interaction reported in labeling; not formally evaluated	Food, not acid suppression, dominates	None specific	L	[20]
Enzalutamide	No	Not pH-dependent; no interaction expected or reported	Not pH-dependent	None specific	M	[11]
Apalutamide	No	Not ionizable across physiological pH	Not pH-sensitive; the same is true of enzalutamide	None specific	M	[12]
Palbociclib	Minimal (fed)	Rabeprazole under fed conditions: Cmax ↓ 41%, AUC ↓ 13% (capsule study)	Contrast to pH-sensitive TKIs	No dose change; take tablet per label	PK	[43]
Ribociclib	No	No interaction reported in labeling; not formally evaluated	Not a primary concern	None specific	L	[37]
Abemaciclib	No	No interaction reported in labeling; not formally evaluated	Not a primary concern	None specific	L	[33]
Olaparib	No major	No interaction reported in labeling; not formally evaluated	CYP3A interactions dominate	None specific	L	[40]
Niraparib	No	Not ionizable across the physiological pH range; no labeled interaction	Carboxylesterase metabolism; pH-insensitive	None specific	M	[38]
Ibrutinib	No (not primary)	No labeled dose change; interaction not formally evaluated	CYP3A is the dominant axis	None specific	L	[54]
Acalabrutinib	Yes	Omeprazole ↓ AUC 43%; antacid ↓ AUC 53%	Within-class divergence from ibrutinib—textbook contrast	Avoid PPIs; stagger H2RAs and antacids ≥ 2 h	PK	[55]
Venetoclax	No (not primary)	No interaction reported in labeling; not formally evaluated	CYP3A/P-gp dominate	None specific	L	[28]
Alectinib	Insensitive to pH	No clinically meaningful effect of esomeprazole	Counterexample: no significant ARA interaction	No dose change	PK	[30,31]
Everolimus	No (not primary)	No interaction reported in labeling; not formally evaluated	CYP3A and P-gp are the dominant axes	None specific	L	[41]

PPI, proton pump inhibitor; H2RA, histamine H2-receptor antagonist; ARA, acid-reducing agent; SmPC, summary of product characteristics; AR, androgen receptor; AUC, area under the concentration–time curve; Cmax, maximum concentration; CYP, cytochrome P450; P-gp, P-glycoprotein; TKI, tyrosine kinase inhibitor. ↓, decrease. Evidence descriptors (PK, L, M and M/PK) are defined in the footnote to Table 1 and in Section 2. Because the magnitude of an acid-suppression interaction depends on dissolution behavior, each entry applies to the formulation examined in the cited source and should not be transferred without qualification to a different product of the same active substance.

## 7. CYP-Mediated and Transporter-Mediated Drug–Drug Interactions

The disposition of most oral anticancer agents is dominated by CYP3A4, and this single fact accounts for the majority of clinically important pharmacokinetic drug–drug interactions in the class [2]. Because CYP3A4 is expressed in both the intestinal wall and the liver (Figure 2, sites 4 and 5), inhibition or induction affects presystemic extraction as well as systemic clearance, and the magnitude of the resulting exposure change can be large for agents with high first-pass metabolism.

As in Section 6, the entries in Table 3 differ in the strength of the evidence behind them, and that difference is made explicit there. Most of the exposure changes quoted below derive from dedicated clinical interaction studies using strong index perpetrators—ketoconazole or itraconazole for inhibition, rifampin for induction—performed because in vitro characterization predicted a clinically relevant effect; this staged, risk-based approach is the current regulatory expectation [7]. A smaller number of entries are model-based projections rather than administered studies, and they are identified as such; the predicted ketoconazole effect on abemaciclib described below is one example, and it is coded in Table 3 as a projection rather than as a measured result. Conversely, where a label records no major interaction, the corresponding entry states the absence of a labeled finding rather than a demonstrated absence of effect; for an agent known to be a substrate, inhibitor or inducer of cytochrome P450 enzymes or of clinically relevant transporters, the mechanistic potential remains, and such an entry should be read as an untested rather than an excluded interaction.

### 7.1. CYP3A4 Inhibition

Strong CYP3A4 inhibitors, which include the azole antifungals ketoconazole and itraconazole, macrolides such as clarithromycin, and several protease inhibitors, reduce clearance and increase the exposure of substrate agents, sometimes dramatically. The abemaciclib label notes that ketoconazole is predicted to increase abemaciclib AUC up to 16-fold [33,60]. A dedicated study of ibrutinib found that ketoconazole increased ibrutinib Cmax and AUC by approximately 29-fold and 24-fold, respectively [54,61]. The magnitude can convert into an outright contraindication, and for venetoclax in chronic lymphocytic leukemia, concomitant strong CYP3A4 inhibitors are contraindicated at initiation and during the dose ramp-up because increased exposure raises the risk of tumor lysis syndrome, with ritonavir shown to increase venetoclax AUC 7.9-fold [28,62]. Where co-administration is unavoidable, labels specify defined reductions, with pazopanib reduced to 400 mg in the presence of a strong inhibitor, olaparib to 100 mg twice daily, palbociclib to 75 mg daily, and venetoclax by at least 75% after ramp-up [23,40,43]. Everolimus shows a similarly large effect, with ketoconazole increasing exposure approximately 15-fold [41].

### 7.2. CYP3A4 Induction

Strong inducers such as rifampin, carbamazepine, phenytoin and St. John’s wort accelerate clearance and reduce exposure, and the effect is frequently large: rifampin reduced exposure by approximately 80% for nilotinib, 85% for palbociclib, 87% for olaparib, and roughly 10-fold for ibrutinib; carbamazepine reduced lapatinib AUC by approximately 72% [40,43,44,52]. Because the ability to compensate by dose escalation is agent-dependent and not always supported, avoidance is generally recommended. A subset of inducers warrants particular attention because of the magnitude of their effect, since cobimetinib exposure falls by approximately 83% with a strong CYP3A inducer and by 73% with a moderate one [53], while rifampin lowers everolimus AUC by 64% [41].

### 7.3. Perpetrator Role of Androgen-Axis Antagonists

Two of the agents reviewed here are themselves strong CYP3A4 inducers, and this perpetrator role is at least as clinically important as their victim profile. Enzalutamide, at steady state in patients, behaved as a strong CYP3A4 inducer in a phenotypic cocktail study, reducing midazolam exposure substantially [11], and apalutamide produced even more dramatic effects in a dedicated drug-interaction study: midazolam AUC fell by 92%, omeprazole AUC by 85%, and S-warfarin AUC by 46% [12]. The implication is that patients on enzalutamide or apalutamide require active management not only of the androgen receptor (AR) antagonist’s own pharmacokinetics but, more importantly, of the wide range of co-medications they will render less effective—statins, anticoagulants, anticonvulsants, immunosuppressants, opioids, and many others metabolized by CYP3A4 or CYP2C19. This pattern—the cancer drug as a drug–drug interaction (DDI) source rather than target—is easily missed by interaction-screening tools focused on the cancer drug’s clearance.

### 7.4. Transporter-Mediated Interactions

Beyond metabolism, the efflux transporters P-gp and BCRP and the uptake transporter family OATP mediate interactions for agents that are their substrates or inhibitors [19,63]. Several agents reviewed here are not only substrates but inhibitors: lapatinib increased the AUC of oral digoxin approximately 2.8-fold; ibrutinib inhibits P-gp and BCRP; venetoclax is both a substrate and an inhibitor of P-gp and BCRP, with the label advising separation of narrow-index P-gp substrates by at least six hours; and pazopanib inhibits UGT1A1 and OATP1B1 [23,28,52,54]. A distinct transporter phenomenon deserves mention because it is easily mistaken for toxicity: inhibition of the renal transporters organic cation transporter 2 (OCT2) and multidrug and toxin extrusion (MATE) can raise serum creatinine without a true fall in glomerular filtration, as characterized for tucatinib [64]; niraparib, which also inhibits MATE1 and MATE2K, may produce analogous transporter-mediated apparent creatinine elevations [38]. An isolated creatinine rise on either agent should prompt consideration of a transporter effect rather than automatic dose reduction. Because transporter and CYP3A4 substrate specificities frequently overlap, a single interacting drug may act through more than one mechanism [65].

The caution stated for food effects in Section 5 applies with equal force to the magnitudes in Table 3. Interaction studies differ in the index perpetrator used and in its dose and duration, in whether the victim drug was given as a single dose or at steady state, in the population studied, and in the endpoint reported, so a larger reported fold-change does not by itself establish that one agent is more interaction-prone than another. The values are best used as an indication of the order of magnitude of the effect for the agent concerned, and of the management response that it requires. Table 3 summarizes the principal interactions for each agent.

**Table 3:** Clinically important pharmacokinetic drug–drug interactions of the twenty-seven oral anticancer agents.

Drug	Major Pathway	Interacting Drug/Class	Expected Exposure Change	Clinical Recommendation	Evidence	Refs.
Imatinib	CYP3A4 substrate; CYP3A4 inhibitor	Ketoconazole/rifampin; simvastatin	Ketoconazole ↑ Cmax/AUC 26%/40%; rifampin ↓ AUC 68%; simvastatin AUC ↑ 3.5×	Caution with strong inhibitors; increase dose by ≥50% with a strong inducer	PK	[8]
Nilotinib	CYP3A4 substrate; P-gp substrate/inhibitor	Ketoconazole/rifampin	Ketoconazole ↑ AUC ~3×; rifampin ↓ ~80%	Avoid strong inhibitors (QT) and inducers; reduce dose if unavoidable	PK	[44]
Dasatinib	CYP3A4 substrate	Strong CYP3A4 inhibitors/inducers	Inhibitors ↑; inducers ↓ (magnitude not reported in the cited source)	Strong inhibitor: reduce dose (100 → 20 mg; 140 → 40 mg); avoid St John’s wort	L	[39,51]
Erlotinib	CYP3A4 (and CYP1A2) substrate	Ketoconazole/rifampin/ciprofloxacin	Ketoconazole ↑ ~67%; rifampin ↓ 58–80%; ciprofloxacin ↑ 39%	Avoid strong inhibitors/inducers; note that smoking ↓ exposure	PK	[24]
Osimertinib	CYP3A4 substrate; weak BCRP/P-gp inhibitor	Rifampin	Strong CYP3A inducer ↓ exposure	Avoid strong inducers; if unavoidable ↑ to 160 mg	L	[36]
Lapatinib	CYP3A4/5 substrate; inhibitor of CYP3A4, CYP2C8, P-gp	Ketoconazole/carbamazepine; digoxin	Carbamazepine ↓ AUC ~72%; digoxin AUC ↑ ~2.8×	Avoid strong inhibitors/inducers; monitor digoxin	PK	[52]
Pazopanib	CYP3A4 substrate; P-gp/BCRP substrate; UGT1A1/OATP1B1 inhibitor	Ketoconazole/rifampin	Ketoconazole ↑ AUC 1.7×	Avoid strong inhibitors; if unavoidable, reduce to 400 mg	PK	[23]
Sunitinib	CYP3A4 substrate	Ketoconazole/rifampin	Ketoconazole ↑ AUC 51%; rifampin ↓ AUC 46%	Reduce dose with a strong inhibitor (37.5 mg GIST/RCC; 25 mg pNET)	PK	[35]
Regorafenib	CYP3A4 + UGT1A9	Ketoconazole/rifampin	Ketoconazole ↑ AUC 33%; rifampin ↓ AUC 50% (M-5 ↑ 264%)	Avoid strong inhibitors/inducers; striking metabolite shift	PK	[32]
Cabozantinib	CYP3A4 substrate; P-gp inhibitor	Ketoconazole/rifampin	Ketoconazole ↑ AUC 38%; rifampin ↓ AUC 77%	Avoid strong inhibitors/inducers; if unavoidable, reduce by 20 mg	PK	[25]
Vemurafenib	CYP3A4 substrate; inhibitor of CYP1A2 and CYP3A4	Itraconazole/rifampin; tizanidine	Itraconazole ↑ AUC 40%; rifampin ↓ AUC 40%; tizanidine AUC ↑ 4.7×	Avoid strong inhibitors/inducers; CYP1A2 substrate exposures rise	PK	[22]
Dabrafenib	CYP2C8/CYP3A4 substrate; CYP3A4/2C9 inducer	Ketoconazole/gemfibrozil/rifampin; midazolam	Ketoconazole ↑ AUC 71%; gemfibrozil ↑ 47%; rifampin ↓ 34%; midazolam ↓ 74%	Avoid strong CYP3A/2C8 inhibitors and inducers; warn regarding CYP3A substrates	PK	[26]
Trametinib	Hydrolytic esterases (not CYP)	No major drug interactions identified	Not significantly affected by CYP inhibitors or inducers	No PK-based dose adjustments	M	[27]
Cobimetinib	CYP3A substrate	Itraconazole/rifampin	Itraconazole ↑ AUC 6.7×; strong inducer ↓ 83%	Avoid strong and moderate inhibitors; if a short-term moderate CYP3A inhibitor is unavoidable, reduce to 20 mg	PK	[53]
Abiraterone	CYP3A4 substrate; strong CYP2D6 inhibitor	Dextromethorphan	Dextromethorphan AUC ↑ ~2.9×	Avoid narrow-therapeutic-index CYP2D6 substrates	PK	[20]
Enzalutamide	CYP2C8 substrate; strong CYP3A4 inducer	Gemfibrozil/rifampin; CYP3A substrates	Gemfibrozil ↑ AUC 2.2×; rifampin ↓ AUC 37%; substantial ↓ of co-medications	Avoid strong CYP2C8 inhibitors; warn regarding CYP3A/2C9/2C19 substrates	PK	[11]
Apalutamide	CYP3A/2C8 substrate; strong CYP3A4/CYP2C19 inducer	Ketoconazole; midazolam, omeprazole, S-warfarin	Ketoconazole ↑ steady-state AUC 51%; midazolam ↓ AUC 92%; omeprazole ↓ 85%; S-warfarin ↓ 46%	Major perpetrator on CYP3A/2C19/UGT substrates	PK	[12]
Palbociclib	CYP3A + SULT2A1; weak CYP3A inhibitor	Itraconazole/rifampin; midazolam	Itraconazole ↑ ~87%; rifampin ↓ ~85%; midazolam ↑ 61%	Avoid strong inhibitors (or reduce to 75 mg); avoid inducers	PK	[43]
Ribociclib	CYP3A4 substrate; moderate CYP3A inhibitor	Ritonavir/rifampin	Ritonavir ↑ AUC 3.2×	Avoid strong inhibitors/inducers; if unavoidable, reduce to 400 mg	PK	[37]
Abemaciclib	CYP3A4 (active metabolites)	Ketoconazole/clarithromycin/rifampin	Ketoconazole predicted ↑ up to 16-fold (model-based); rifampin ↓ 67%	Avoid ketoconazole; other strong inhibitors: reduce to 100 mg twice daily	M/PK	[33,60]
Olaparib	CYP3A	Itraconazole/fluconazole/rifampin	Itraconazole ↑ 170%; fluconazole ↑ 121%; rifampin ↓ 87%	Avoid strong and moderate inhibitors; if unavoidable, reduce (strong inhibitor → 100 mg twice daily)	PK	[40,63]
Niraparib	Carboxylesterases + UGT (not CYP3A)	No significant CYP interactions identified	Not affected by CYP3A inhibitors or inducers	No CYP-based dose adjustment; inhibits MATE1/2K (creatinine ↑ possible)	M	[38]
Ibrutinib	CYP3A substrate; P-gp/BCRP inhibitor	Ketoconazole/rifampin; grapefruit	Ketoconazole ↑ Cmax/AUC ~29×/24×; rifampin ↓ ~10×	Avoid strong inhibitors; moderate inhibitor → 140 mg; avoid grapefruit and inducers	PK	[54,61]
Acalabrutinib	CYP3A substrate; weak CYP3A4 inducer	Itraconazole/rifampin	Itraconazole ↑ Cmax/AUC 3.9×/5.1×; rifampin ↓ Cmax/AUC 68%/77%	Avoid strong inhibitors and inducers; severe hepatic impairment: avoid	PK	[55]
Venetoclax	CYP3A4/5; P-gp and BCRP substrate/inhibitor	Strong CYP3A inhibitors; ritonavir; P-gp substrates	Ritonavir ↑ AUC 7.9×	Contraindicated with a strong inhibitor at ramp-up (CLL/SLL); after ramp-up, reduce by ≥75%	PK	[28,62]
Alectinib	CYP3A4 to active metabolite M4	Posaconazole/rifampin	No clinically meaningful effect on alectinib + M4	No FDA adjustment; EMA recommends monitoring with a strong inducer	PK	[30,66]
Everolimus	CYP3A4 substrate; P-gp substrate/inhibitor	Ketoconazole/erythromycin/rifampin	Ketoconazole ↑ AUC 15×; erythromycin ↑ 4.4×; rifampin ↓ AUC 64%	Avoid strong inhibitors; reduce dose with a moderate inhibitor	PK	[41]

BCRP, breast cancer resistance protein; MATE, multidrug and toxin extrusion; OATP, organic anion transporting polypeptide; P-gp, P-glycoprotein; SULT, sulfotransferase; UGT, uridine diphosphate glucuronosyltransferase; CLL/SLL, chronic lymphocytic leukemia/small lymphocytic lymphoma; GIST, gastrointestinal stromal tumor; RCC, renal cell carcinoma; pNET, pancreatic neuroendocrine tumor; AUC, area under the concentration–time curve; Cmax, maximum concentration; CYP, cytochrome P450; EMA, European Medicines Agency; FDA, Food and Drug Administration. ↑, increase; ↓, decrease. Evidence descriptors (PK, L, M and M/PK) are defined in the footnote to Table 1 and in Section 2.

## 8. Exposure–Response, Exposure–Toxicity, and Therapeutic Drug Monitoring

The clinical importance of the pharmacokinetic perturbations described above derives from the fact that, for many oral anticancer agents, systemic exposure is related to outcome. Where an exposure–response relationship exists, reduced exposure risks diminished antitumor effect; where an exposure–toxicity relationship exists, increased exposure risks dose-limiting adverse effects. For several agents both relationships have been described.

Some exposure-related toxicities are characteristic of the class and inform monitoring. Tyrosine kinase inhibitors have been associated with metabolic and endocrine complications and with elevations in serum creatine kinase [67,68]. These relationships are also the rationale for interest in therapeutic drug monitoring (TDM). For a subset of oral anticancer agents, plasma concentration targets associated with efficacy or with acceptable toxicity have been proposed, and structured protocols for routine TDM have been developed [6]. A particularly concrete example is everolimus in subependymal giant cell astrocytoma associated with tuberous sclerosis complex, where the label specifies a whole-blood trough target of 5–10 ng/mL and titrates the dose accordingly [41]—a labeled, prospective use of TDM that stands somewhat apart from the more variable evidence base for other agents. Candidate scenarios in which TDM is most plausibly useful include suspected non-adherence, unexplained toxicity or lack of response, known or suspected drug interactions, organ impairment, and populations underrepresented in registration trials; venetoclax and pazopanib are among the agents for which TDM has been specifically explored [15,16].

The evidence base for TDM in oral oncology is uneven and should not be overstated. Routine, unselected monitoring is not supported by current evidence and is not recommended here. The clearest exception is the small group of situations in which a labeled concentration target exists and titration against it forms part of the approved use, of which everolimus in subependymal giant cell astrocytoma is the example given above [41]. Exposure targets proposed for other agents remain investigational [6,15,16], and monitoring outside such protocols is best reserved for the defined clinical questions listed above, where a concentration measurement can change a decision that would otherwise be made without information. For most agents the relationship between concentration and outcome is insufficiently defined to support monitoring of any kind, and limited assay availability, turnaround time and the absence of validated targets remain practical constraints.

## 9. Special Populations and Patient-Level Factors

The exposure achieved by a standard dose varies systematically across patient subgroups, several of which are common in the oncology population. Because hepatic metabolism dominates the clearance of most oral anticancer agents, impaired hepatic function frequently increases exposure: ibrutinib AUC rises approximately 2.7-, 8.2-, and 9.8-fold in mild, moderate, and severe hepatic impairment respectively, and the agent is not recommended in moderate-to-severe impairment [54]; venetoclax AUC is approximately 2.7-fold higher in severe impairment, prompting a labeled dose reduction [28,69]; and palbociclib is reduced to 75 mg daily in severe (Child-Pugh C) impairment [43]. Renal elimination of unchanged drug is minor for many oral targeted agents but is not universally so—olaparib, for example, carries a labeled dose reduction in moderate renal impairment and has not been formally evaluated in severe impairment [40].

Advanced age aggregates reduced organ-function reserve, higher prevalence of polypharmacy, and altered body composition; the probability of a clinically significant interaction rises with the number of concomitant medications, and altered gastrointestinal anatomy after surgical resection or in malabsorptive states can change the surface area, pH, transit, and transporter expression on which absorption depends [3]. Heritable variation in metabolizing enzymes and transporters contributes to exposure variability for selected agents—the lapatinib label, for instance, links the human leukocyte antigen (HLA) alleles DQA1*02:01 and DRB1*07:01 to an elevated risk of hepatotoxicity [52]—though pharmacogenomic dosing guidance is not established for most oral anticancer agents. The determinant unique to oral therapy is adherence: imperfect adherence reduces effective exposure independently of every pharmacokinetic property discussed above.

## 10. Implications for Precision Oncology Pharmacy Practice

The pharmacokinetic principles assembled above acquire clinical value only when they are operationalized at the point of care, and the oncology pharmacist is positioned to do precisely this [3]. Pharmacokinetic optimization is not a single intervention but a sequence of linked assessments performed at initiation and revisited at each clinically relevant change.

At initiation, medication reconciliation establishes the complete medication list against which interaction screening is performed. Screening should explicitly include the recurring CYP3A4 and transporter culprits and, critically, acid-suppressive therapy, which patients frequently omit from a medication history [5]. Meal-timing counseling translates the agent’s food-effect profile into concrete instruction—including the interval relative to meals [46]—with verification that the instruction is understood. Assessment of hepatic and renal function identifies the need for dose adjustment before the first dose. During therapy, the pharmacist monitors for toxicity, supports dose modification, reassesses adherence, and re-screens for interactions whenever a medication is added or stopped.

Figure 1 presents this work as a structured framework that proceeds through eight steps, beginning with confirmation of the agent and dosing schedule and continuing through assessment of meal requirements, screening for acid-suppressive therapy, screening for cytochrome P450 and transporter interactions, assessment of organ function and special-population factors, evaluation of toxicity and exposure-related concerns, patient-specific counseling with an accompanying monitoring plan, and reassessment after any medication change, episode of toxicity or change in disease status. The eighth step returns to the first, rendering the framework cyclical rather than linear.

## 11. Limitations, Knowledge Gaps, and Future Directions

The twenty-seven agents profiled in detail represent a deliberate mechanistic sample, as Section 2 explains, drawn from a larger and rapidly growing group of agents that now exceeds one hundred FDA-approved oral oncology molecules. The boundary of the present sample is drawn most sharply at recency: a substantial cohort of oral targeted therapies has been approved since 2023 that does not appear in the tables, including the menin inhibitor revumenib for relapsed or refractory acute leukemia [70]; the MEK1/2 inhibitor mirdametinib for NF1-associated plexiform neurofibromas [71]; the avutometinib–defactinib co-pack for KRAS-mutated low-grade serous ovarian cancer [72]; the oral selective estrogen receptor degrader imlunestrant for ESR1-mutated advanced or metastatic breast cancer [73]; and three new kinase inhibitors directed at human epidermal growth factor receptor 2 (HER2) or epidermal growth factor receptor (EGFR) for non-small-cell lung cancer—zongertinib (HER2 tyrosine kinase domain) [74], sevabertinib (HER2/EGFR) [75], and sunvozertinib (EGFR exon 20 insertion) [76]. Their labeling illustrates that the phenomena described in this review continue to govern newly approved agents: imlunestrant is to be taken at least two hours before or one hour after food because a low-fat meal approximately doubles its exposure [73]; sunvozertinib and sevabertinib are each to be taken with food [75,76]; revumenib is administered fasted or with a low-fat meal [70]; avutometinib and defactinib are each taken with food [72]; and zongertinib, although unaffected by food, requires avoidance of strong CYP3A inducers, carbamazepine having reduced its exposure by 63% [74]. These agents and others approved in the same window were considered for inclusion but were excluded for two reasons: their labeled pharmacokinetic profiles in most cases either parallel an included agent of the same class (so they would expand the table without exposing a new mechanistic lesson) or are still maturing in the post-marketing literature. They are nonetheless clinically important, and the present review should be interpreted as illustrating principles whose application extends to these and to subsequent approvals, not as cataloguing every oral anticancer agent in current use.

Several limitations constrain the precision with which oral anticancer therapy can be individualized, and some of them belong to this review rather than to the field. Agent selection, although governed by the criteria set out in Section 2, involved judgement at the margin, so a different author applying the same criteria could reasonably have profiled a partly different set of twenty-seven agents. The deliberate exclusion of close within-class analogues means that Table 1, Table 2 and Table 3 are not a census of labeled pharmacokinetic effects and are not reproducible in the sense that a systematic review would be, and no formal risk-of-bias appraisal was applied to the included studies, so the evidence descriptors record the type of evidence supporting each entry rather than its methodological quality. Turning from the review to the evidence it summarizes, several further limitations apply. Food-effect studies are heterogeneous in design [1,9], which complicates cross-agent comparison and the translation of registration-study conditions to ordinary eating behavior. Real-world pharmacokinetic data remain limited, so the exposure variability actually experienced in practice is incompletely characterized. Special populations are underrepresented in the trials that establish dosing, leaving their management dependent on extrapolation. The integration of pharmacogenomics into oral oncology dosing remains partial. Therapeutic drug monitoring, despite a sound rationale for selected agents, is constrained by incomplete exposure–response characterization and limited assay infrastructure [6]. Finally, pharmacist-led pharmacokinetic optimization is itself under-studied as an intervention; prospective evaluation of its effect on exposure, toxicity, adherence, and outcome would strengthen the evidentiary basis for the practice model proposed here.

## 12. Conclusions

Oral anticancer agents have expanded the reach and convenience of cancer treatment, but they have done so by making systemic exposure dependent on a chain of absorption and disposition processes that intravenous therapy bypasses. Food intake, gastric pH and acid-suppressive therapy, CYP3A4- and transporter-mediated drug interactions, hepatic and renal function, and adherence can each shift exposure to a clinically meaningful degree, and the narrow therapeutic margins of many of these agents convert such shifts into differences in toxicity and effect. Individualized pharmacokinetic assessment is therefore not an academic refinement but a practical requirement of safe and effective oral anticancer therapy. The oncology pharmacist, applying a structured and repeated framework of reconciliation, interaction and acid-suppression screening, meal-timing counseling, organ-function assessment, and toxicity monitoring, is well placed to deliver this assessment and to realize the promise of precision in everyday oncology practice.

## Data Availability

No new data were created or analyzed in this study. All data referenced are available in the cited publications and product labels.

## Abbreviations

The following abbreviations are used in this manuscript:

ALK	anaplastic lymphoma kinase
ARA	acid-reducing agent
AUC	area under the concentration–time curve
BCL-2	B-cell lymphoma 2
BCRP	breast cancer resistance protein (ABCG2)
BCS	Biopharmaceutics Classification System
BID	twice daily
BTK	Bruton tyrosine kinase
CDK	cyclin-dependent kinase
CLL	chronic lymphocytic leukemia
Cmax	maximum plasma concentration
CYP	cytochrome P450
DDI	drug–drug interaction
EGFR	epidermal growth factor receptor
EMA	European Medicines Agency
F	oral bioavailability
FDA	Food and Drug Administration
GI	gastrointestinal
H2RA	histamine H2-receptor antagonist
HER2	human epidermal growth factor receptor 2
HLA	human leukocyte antigen
MATE	multidrug and toxin extrusion
OATP	organic anion transporting polypeptide
OCT	organic cation transporter
PARP	poly(ADP-ribose) polymerase
P-gp	P-glycoprotein (ABCB1)
PK	pharmacokinetics
PPI	proton pump inhibitor
SmPC	summary of product characteristics
TDM	therapeutic drug monitoring
TKI	tyrosine kinase inhibitor
TLS	tumor lysis syndrome
UGT	uridine diphosphate glucuronosyltransferase
VEGFR	vascular endothelial growth factor receptor
